# KidsBrainIT: Visualization of the Impact of Cerebral Perfusion Pressure Insult Intensity and Duration on Childhood Brain Trauma Outcome

**DOI:** 10.1007/s12028-025-02296-z

**Published:** 2025-06-03

**Authors:** Bavo Kempen, Bart Depreitere, Ian Piper, Maria Poca, Stefan Mircea Iencean, Mireia Garcia, James Weitz, Gayathri Subramanian, Roddy O’Kane, Julian Zipfel, Arta Barzdina, Stefano Pezzato, Patricia A. Jones, Tsz-Yan Milly Lo, the KidsBrainIT consortium

**Affiliations:** 1 Department of Neurosciences, KU Leuven, Leuven, Belgium; 2 Department of Neurosurgery, University Hospitals Leuven, Leuven, Belgium; 3 Usher Institute, University of Edinburgh, Edinburgh, UK; 4 Paediatric Critical Care Unit, Royal Hospital for Children & Young People, Edinburgh, UK; 5 Department of Neurosurgery, Vall d’Hebron University Hospital, Barcelona, Spain; 6 Department of Neurosurgery, GrT Popa University of Medicine and Pharmacy, Iasi, Romania; 7 Paediatric Intensive Care Unit, Bristol Royal Hospital for Children, University Hospitals Bristol NHS Foundation Trust, Bristol, UK; 8 Paediatric Intensive Care, Parc Taulí University Hospital, Sabadell, Spain; 9 Paediatric Critical Care, John Radcliffe Hospital, Oxford University Hospitals NHS Foundation Trust, Oxford, UK; 10 Paediatric Intensive Care Unit, Royal Manchester’s Children’s Hospital, Manchester University Hospitals NHS Foundation Trust, Manchester, UK; 11 Royal Hospital for Children & Institute of Neurological Sciences, Glasgow Paediatric Neurosurgery, Goven Road, Glasgow, UK; 12 Section of Pediatric Neurosurgery, Department of Neurosurgery, University Hospital Tübingen, Tübingen, Germany; 13 Clinic for Anesthesiology and Intensive Care, Children’s Clinical University Hospital, Riga, Latvia; 14 Neonatal and Pediatric Intensive Care Unit, IRCCS Istituto Giannina Gaslini, Genova, Italy; 15 Child Life and Health, University of Edinburgh, Edinburgh, UK

**Keywords:** Cerebral perfusion pressure, Child, Cerebrovascular reactivity, Dose–response plot, Intracranial pressure, Traumatic brain injury

## Abstract

**Background:**

Cerebral perfusion pressure (CPP) dose–response on post–traumatic brain injury (TBI) outcome in children remains unknown. This project aimed to produce the first pediatric post-TBI CPP dose–response visualization plot from the international multicenter KidsBrainIT data set.

**Methods:**

Fully anonymized prospectively collected routine minute-by-minute intracranial pressure (ICP), mean arterial blood pressure, and CPP time series data from 104 pediatric patients with TBI were categorized into CPP intensity duration episodes, albeit CPP above or below a range of thresholds. These episodes were then correlated with the 6-month modified Glasgow Outcome Score (GOS) and depicted in 3D color-coded CPP dose–response plots. Additionally, the effects of cerebrovascular reactivity patterns and ICP were examined.

**Results:**

Our pediatric CPP dose–response plots resembled the previously published adult CPP dose–response plots: on the CPP pressure time plots, an exponential “black” transition curve separated CPP episodes associated with poor (“red,” GOS < 4) and good (“blue”) outcome. Lower and higher ends of CPP intensity were only tolerated for shorter durations. A “safe” CPP zone (56–89 mm Hg) was identified for childhood TBI with active cerebrovascular reactivity pattern and ICP < 20 mm Hg. Passive cerebrovascular reactivity pattern reduced the area of safe CPP doses. ICP levels > 20 mm Hg were associated with worse outcome, irrespective of CPP dose.

**Conclusions:**

The pediatric CPP dose–response on poor outcome was visualized successfully for the first time. Because the “critical” lower CPP limit exceeds the current recommended minimum CPP target for pediatric TBI treatments, there is an urgent need to validate childhood CPP dose–response to provide evidence-based CPP clinical targets in the future.

**Supplementary Information:**

The online version contains supplementary material available at 10.1007/s12028-025-02296-z.

## Introduction

Pediatric traumatic brain injury (TBI) is a global health challenge affecting a diverse demographic of more than 3 million children worldwide [1–3]. Although severe brain trauma accounts for only up to 7% of pediatric TBI cases, it remains a leading cause of death and acquired disability among survivors despite improved trauma and critical care [2]. Childhood TBI has a long-lasting increased associated socioeconomic cost per individual due to its impact on executive functions and memory formation, which are essential for schooling and social success [2, 4, 5].

Minimizing additional secondary physiological insult to the already injured brain remains the mainstay of modern severe TBI management for both adults [6] and children [7]. Raised intracranial pressure (ICP), reduced cerebral perfusion pressure (CPP), hypotension, and hypoxia are known physiological insults associated with morbidity and mortality in severe TBI [8–15]. Thus, modern TBI management guidelines [6, 7] emphasize the prevention, early detection, and prompt treatment of raised ICP with optimization of CPP to safeguard adequate cerebral blood flow because CPP, calculated as the difference between mean arterial blood pressure (mABP) and ICP, is the proxy pressure gradient to drive cerebral blood flow [16].

The Brain Trauma Foundation guidelines recommended monitoring of ICP in severe pediatric patients with TBI to guide treatments, with the most recent guidelines recommending ICP-lowering treatments to be commenced if ICP is raised above 22 mm Hg in adults and 20 mm Hg in pediatric patients for more than 5 min [6, 7, 17]. These ICP treatment threshold recommendations were inspired by the concept of ICP dose, which highlights the relationship between poor neurological outcome and sustained duration of intracranial hypertension in severe TBI [18]. Güiza and other colleagues subsequently demonstrated ICP dose–response in adult and pediatric severe TBI using a 3D color-coded plot to visualize the intuitive concept that the higher the ICP, the shorter the duration a patient with TBI could tolerate before poor neurological outcome [19–21]. Furthermore, a CPP threshold ≤ 50 mm Hg was found to be detrimental to the tolerance of ICP dose–response in both adult and pediatric patients [19, 21].

The post-TBI CPP treatment targets for both adults and children in modern TBI management guidelines are based on level IIB and level III evidence [6, 7]. The latest Brain Trauma Foundation guidelines recommend maintaining CPP between 60 and 70 mm Hg for adults [6], and for pediatric TBI, a target between 40 and 50 mm Hg is recommended to ensure the minimum value (40 mm Hg) is not reached [7]. However, this guidance does not consider the dose–response, which provides better insight into the burden of CPP insults on outcome in terms of severity and duration.

CPP dose–response on outcome in adult patients with TBI was previously visualized by adapting the ICP dose visualization plot methodology [19, 22]. Just like the ICP dose–response plot, the 3D color-coded adult CPP dose–response plot showed an exponential “black” transition curve separating positive (“blue”) and negative (“red”) association of 6-month post-TBI global outcome with CPP insults [22]. Assessing the relationship of CPP events above and below different levels with outcome, a “safe” zone of CPP values was defined and demonstrated both lower and higher CPP values were only tolerated for limited durations [22]. Furthermore, the CPP transitional curves forming the safe zone when selectively visualizing CPP events changed in accordance with active versus passive vasoreactivity pattern or with intracranial hypertension [22]. These adult TBI CPP dose–response analyses provide clinicians with enhanced insight into how CPP insult burden impacted outcome, but CPP dose–response visualization plots have not been reproduced in pediatric brain trauma.

This study aims to produce the CPP dose–response visualization plots for the first time in an exclusive pediatric TBI multicenter cohort.

## Methods

A prospective observational study was conducted through the multinational, multicenter KidsBrainIT consortium.

### Patients and Data

A total of 146 children aged 2 to less than 16 years old who had sustained an accidental TBI and clinically required ICP monitoring and pediatric intensive care unit (PICU) care within the KidsBrainIT consortium were included in the study [23]. Ethical and other regulatory approvals were obtained in all 16 participating PICUs from seven countries (UK, Belgium, Spain, Romania, Latvia, Italy, and Germany). The study was registered with the National Health Service, the National Health Service Research Scotland Permissions Coordinating Centre, and the Health Research Authority. Prior to including each patient’s data into the study, fully informed consent was obtained from the legal guardian. Patients followed standard escalating treatment procedures outlined in the local PICU TBI guidelines, with all contributing PICUs within the KidsBrainIT consortium having similar treatment guidelines.

Throughout their PICU stay, patients had minute-by-minute ICP and arterial blood pressure recorded as part of routine clinical care. A parenchymal probe was used for ICP measurements, and invasive arterial cannulation was performed for arterial blood pressure monitoring, zeroed as per institutional guidelines. Bedside monitors calculated CPP as the difference between mABP and ICP. This information, along with other routinely collected physiological data recorded throughout their entire PICU stay, was extracted for the study. A predesigned clinical research proforma collected demographic and clinical details, including postresuscitation Glasgow Coma Score (GCS), pupillary reactions, etc. [19, 21]. A predesigned telephone questionnaire was used to assign modified Glasgow Outcome Scores (GOS) determining global outcomes at 6 months post injury [11, 12, 19, 21, 24–27].

Out of a total of 146 patients, none had decompressive craniectomy and 104 had all the required data for CPP dose–response analyses. Patients were excluded from the analysis because of missing physiology time series data (*n* = 30) or GOS data (*n* = 12). The assessed data encompassed age, sex, motor GCS, admission pupil reactivity, minute-by-minute physiological ICP and mABP time series, and GOS. Artifact annotation and removal in the physiological data was conducted jointly by a part-time KidsBrainIT researcher and a custom-built automated artifact detection system. Both the KidsBrainIT researcher and the automated artifact detection algorithm were trained using the same standard operating procedure for artifact detection as employed by independent experts in previously reported studies [11, 12, 19, 21, 22].

### Visualization Method

The CPP dose–response plots were produced using the visualization method previously described [22], which was an adaptation of the original ICP dose–response plot [19, 21]. Each time series of minute-by-minute CPP data was first segmented into different episodes called CPP insults, which were defined according to their intensity on the *x*-axis and duration on the *y*-axis. Two types of CPP insults were characterized by the measured CPP either dropping below or remaining above a rolling CPP threshold [22]. The CPP visualization was therefore performed separately for insults of low CPP (CPP_Below_) and insults of high CPP (CPP_Above_).

For each CPP insult, the Pearson correlation coefficient between outcome and the average number of these types of CPP insults per patient in each GOS category was then computed [22]. The correlation coefficient ranged from − 1 to 1 and measured the degree of linear dependency. A negative value indicated that the particular CPP insult occurred more frequently with lower GOS, and a positive correlation indicated that the particular CPP insult was more frequent with higher GOS. A correlation was excluded if the event did not occur in at least 20% of the patient population. A contour plot showing lines of equal correlation was then derived from the grid and color coded with dark red and dark blue respectively representing correlations of − 1 and + 1. The black line highlighted the contour of zero correlation and was defined as the “transition curve” because it indicated the transition into the region of insult types that occurred more frequently in patients with lower GOS. Uncertainty of the CPP_Below_ and CPP_Above_ transition lines was assessed by computing a Fisher transformation for each computed Pearson correlation with an 80% confidence interval (CI) and by population bootstrapping with replacement (± 2 SDs) [21, 28, 29].

### Role of Cerebrovascular Reactivity

The influence of active versus passive cerebrovascular reactivity patterns on the association between CPP events and GOS was assessed using the computed median moving minute-by-minute correlation of prior ICP and mABP values over windows ranging from 3 to 120 min, known as the low-frequency autoregulation index (LAx) [19, 22, 30]. Subsequently, CPP event types were classified into either a pressure active (LAx ≤ 0) or pressure passive (LAx > 0) vasoreactivity [19, 22]. A locally weighted nonparametric regression (LOESS) of the second order with an α of 0.2 was used to smooth the combined CPP_Above_ and CPP_Below_ transition curves.

### Role of ICP

The effect of ICP on the CPP events and outcome association was investigated by visualizing the CPP event plots separately for events associated with average ICP < 20 mm Hg and ICP ≥ 20 mm Hg. The 20-mm Hg ICP threshold was previously associated with poorer global outcome (GOS) in pediatric patients [19, 21].

### Multivariable Analyses

The independent association of the cumulative dose of CPP insult burden with outcome was investigated using multivariable binomial logistic regression models. The cumulative dose of CPP insults was conceptualized by computing the percentage of whole monitoring time a patient spent in the red zone during their entire PICU stay of the constructed CPP event graph. The percentage of time spent in the red zone was conceptualized for (1) CPP_Above_, (2) CPP_Below_, and (3) their average. The model was further populated with the International Mission on Prognosis and Clinical Trial Design in TBI (IMPACT) model core variables as covariates: age, admission pupil reactivity, and admission motor GCS [21, 31–33]. All analyses were performed using Python 3.7.1 and R 4.0.3 [34, 35].

## Results

Table 1 summarizes the patient injury demographics, and Table 2 displays the demographic distribution of admission GCS, age, pupil reactivity, and percentage of valid CPP monitored time. The majority of our cohort had favorable outcome (*n* = 65 with GOS > 3).

**Table 1:** Patient injury demographics

Mechanisms of injury	*N* = 104
*Causes*	
Pedestrian	50
Motor vehicle	15
Bicycle	10
Falls	15
Struck on head	9
Sports	3
Penetrating injury	2
*Initial CT brain findings (> 1 feature allowed)*	
EDH	19
SDH + / − SAH	15
DAI	53
Contusions	24
Other	12

*DAI* diffuse axonal injury, *EDH* evacuated extradural haematoma, *SAH* unevacuated subarachnoid hematoma, *SDH* uunevacuated subdural haematoma, *SAH* unevacuated subarachnoid haematoma, *DAI* diffuse axonal injury

**Table 2:** Patient characteristics summarized along GOS-6 (*n* = 104)

GOS-6	GOS-6				
	1 (*n* = 10)	2 (*n* = 4)	3 (*n* = 25)	4 (*n* = 31)	5 (*n* = 34)
Summated GCS					
Median (IQR)	3.50 (3.00, 5.75)	6.50 (5.25, 7.00)	23; 6.00 (3.00, 7.00)	29; 6.00 (4.00, 9.00)	32; 7.00 (3.00, 9.50)
Motor GCS					
Median (IQR)	9; 1.00 (1.00, 2.00)	2.50 (1.00, 4.00)	21; 1.00 (1.00, 3.00)	23; 3.00 (1.00, 5.00)	25; 2.00 (1.00, 5.00)
Age (Years)					
Median (IQR)	10.50 (4.75, 12.00)	8.50 (3.75, 13.25)	7.50 (5.00, 11.00)	10.33 (6.00, 13.29)	10.00 (6.17, 12.75)
Pupil reactivity, *n* (%)					
None; N (%)	0 (0)	0 (0)	0 (0)	0 (0)	1 (3)
One; N (%)	1 (11)	1 (25)	3 (12)	2 (7)	1 (3)
Two; N (%)	8 (89)	3 (75)	21 (88)	28 (93)	29 (94)
CPP monitored time (days)					
Median (IQR)	3.56 (2.06, 4.44)	3.04 (2.50, 11.67)	4.58 (1.75, 8.40)	4.97 (3.23, 6.76)	3.92 (1.29, 4.81)
% valid CPP monitored time					
Median (IQR)	68.87 (47.25, 90.92)	88.90 (75.08, 95.15)	84.44 (71.08, 96.05)	97.59 (79.31, 99.05)	69.26 (36.54, 96.90)

GOS-6 ranges from 1 (death) to 5 (good outcome). The numbers preceding a semicolon reflect the total amount of patients of the GOS subpopulation thatwho had the respective variable available

*CPP* cerebral perfusion pressure, *GCS* Glasgow Coma Score, *GOS-6* Glasgow Outcome Score at 6 months, *IQR* interquartile range

**Fig. 1:**
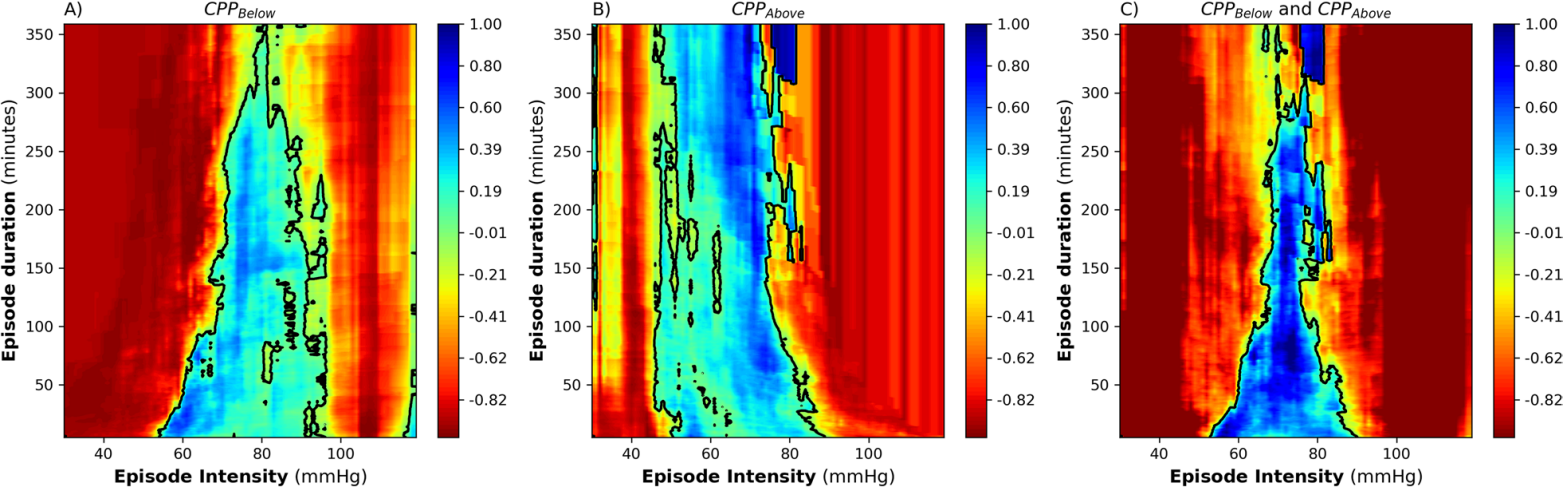
Visualization of the univariate correlation between the Glasgow Outcome Score (GOS) and the average amount of cerebral perfusion pressure (CPP) events per GOS category, *N* = 104. CPP events are denoted by the colored dots in the graph, defined by an intensity threshold (*x*-axis) maintained for a certain duration (*y*-axis). A negative association of a CPP event with GOS is color coded as dark red, which gradually transitions toward dark blue for positive correlations. The zero correlation line, that is, the transition curve, is highlighted in black. **a** CPP_Below_ events, that is, CPP (*x*-axis) represents the threshold defining events where CPP went below this threshold for a certain duration of time. **b** CPP_Above_ events, that is, CPP (*x*-axis) represents the threshold defining events where CPP went above this threshold for a certain duration of time. **c** CPP_Below_ and CPP_Above_ combined

The CPP intensity duration association with GOS visualization is shown in Fig. 1. Both the CPP_Below_ and CPP_Above_ variants of the visualization technique depict a clear blue (CPP events associated with better GOS) and red region (CPP events associated with worse GOS) demarcated by a black transition curve that signals zero correlation. Notably, the CPP visualization techniques (CPP_Below_ and CPP_Above_) pick up signal from the opposite CPP intensity limit to which they were tuned for. For example, CPP_Below_ should emphasize the lower limit on the CPP axis but returned, in addition, an attenuated signal in the higher CPP ranges, reflecting associations between high CPP events and GOS.

In-depth examination of the CPP_Below_ and CPP_Above_ plots revealed that CPP event intensities below 56 mm Hg and above 89 mm Hg were not tolerated and were consistently associated with low GOS scores. In the CPP_Below_ plot, CPP intensities above 56 mm Hg were tolerated for a gradually increasing longer duration. Conversely, in the CPP_Above_ plot, CPP intensities below 89 mm Hg were tolerated for a gradually increasing longer duration. When combining both plots (CPP_Below_ and CPP_Above_), no CPP intensity value is universally associated with good outcome for longer than 306 min, as reflected by the inflection point of the transition curves (Fig. 1c). Graphs were cut off at 360 min from the onset of a specific CPP insult due to the low amount of insults present that lasted longer than 360 min, as shown in previously published work of this methodology in adult TBI [19, 22]. Uncertainty estimates of the CPP_Below_ and CPP_Above_ plots and their transition lines are summarized in Supplementary Figs. 1–4.

**Fig. 2:**
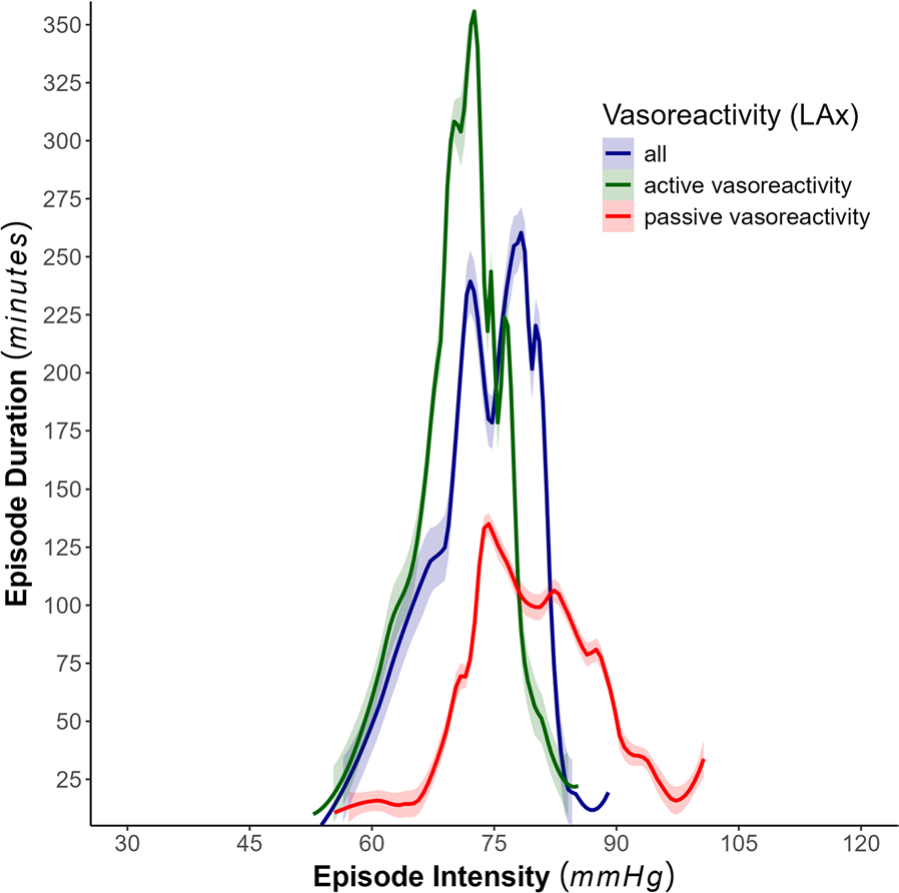
Display of the combined cerebral perfusion pressure (CPP) events transition curves along vasoreactivity state and their 95% confidence interval. Active vasoreactivity reflected in green (low-frequency autoregulation index [LAx] ≤ 0), passive vasoreactivity reflected in red (LAx > 0), and the original transition curves (without selecting for vasoreactivity) reflected in blue. Transition curves of CPP_Below_ and CPP_Above_ were joined and smoothed using locally weighted nonparametric regression (α = 0.2)

On average, in 26.4% (± 29.26%) of the total monitoring time, no LAx could be computed. When LAx was available, patients were governed by active vasoreactivity pattern for 54.5% (± 21.77%) of the monitoring time. In Fig. 2, the combined transition curves for CPP_Below_ and CPP_Above_ were displayed along vasoreactivity patterns after smoothing with LOESS. Under passive vasoreactivity pattern (red), both transition curves (CPP_Below_ and CPP_Above_) shifted to the right on the CPP intensity axis and showed a drastic reduction in maintained event duration associated with good outcome under the triangular curve with respect to active vasoreactivity pattern (green) and combined patterns (blue). The transition curves derived under active vasoreactivity pattern shifted slightly left compared with the combined vasoreactivity patterns. However, durations up to 360 min could be maintained for a negligible range of CPP intensities.

**Fig. 3:**
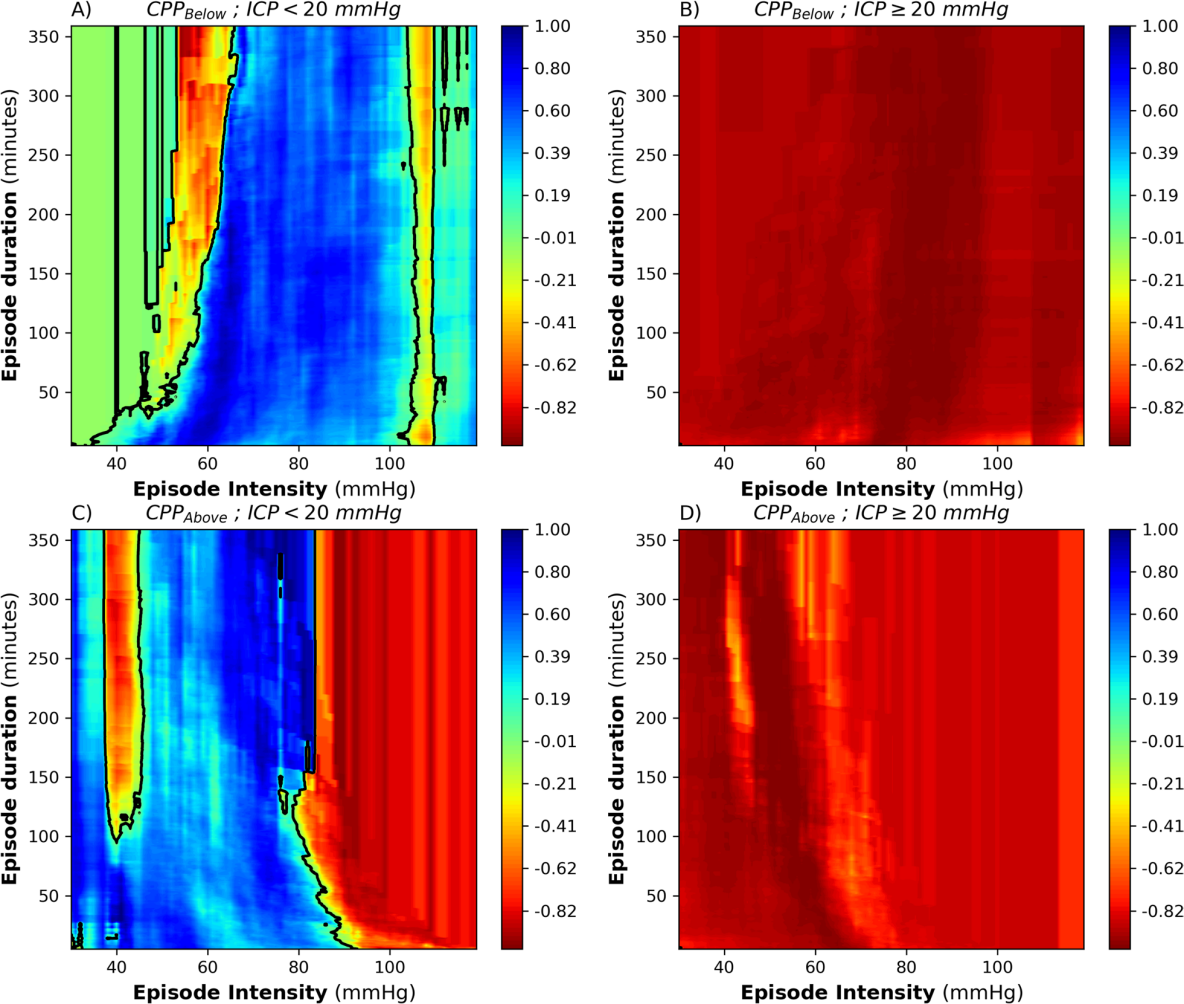
Visualization of the univariate correlation between the Glasgow Outcome Score (GOS) and the average amount of cerebral perfusion pressure (CPP) events per GOS category, delineated by an intracranial pressure (ICP) threshold of 20 mm Hg. The red and blue color-coded zones denote a negative and positive correlation of CPP events with GOS, with CPP event intensity (*x*-axis) and duration (*y*-axis). Zero correlation contour is highlighted in black. **a** CPP_Below_ with ICP < 20 mm Hg. **b** CPP_Below_ with ICP ≥ 20 mm Hg. **c** CPP_Above_ with ICP < 20 mm Hg. **d** CPP_Above_ with ICP ≥ 20 mm Hg

Next, the CPP_Below_ and CPP_Above_ visualizations were repeated but solely for CPP events with either ICP < 20 mm Hg or ICP ≥ 20 mm Hg, as shown in Fig. 3. When ICP was below 20 mm Hg, CPP doses with intensities between 30 and 93 mm Hg were better tolerated for increasing durations (Fig. 3a, c). Conversely, when the CPP_Below_ and CPP_Above_ plots were visualized for events with ICP ≥ 20 mm Hg only, the plots turned completely, red irrespective of CPP insult intensity and duration (Fig. 3b, d).

On average, patients spent 53.28% (± 12.9%) of their total monitoring time in the red zone. Taken together with the IMPACT covariates (age, pupil reactivity, GCS motor) in a multivariable logistic regression, the time spent in the red zone was an independent predictor of mortality (GOS 1) (odds ratio [OR] 1.07; 95% CI 1.01–1.16; *p* = 0.035) but not of unfavorable neurological outcome (GOS < 4) (OR 1.03; 95% CI 1.0–1.08; *p* = 0.1) (Table 3). Motor GCS was a significant predictor for unfavorable neurological outcome (OR 0.71; 95% CI 0.52–0.95; *p* = 0.025). The results of the latter analyses for solely the CPP_Above_ and CPP_Below_ plots are summarized in Supplementary Tables 1 and 2, respectively.

**Table 3:** Average time in red zone of CPP_Aabove_ and CPP_Bbelow_

Characteristic	GOS unfavourable			GOS mortality		
	OR	95% CI	*p*-value	OR	95% CI	*p*-value
Proportion monitoring time in red zone	1.03	1.0, 1.08	0.10	1.07	1.01, 1.16	0.035
Pupil reactivity	1.26	0.32, 5.20	0.7	0.82	0.04, 4.79	0.9
Motor GCS	0.71	0.52, 0.95	0.025	0.67	0.31, 1.10	0.2
Age (years)	1.00	0.88, 1.13	> 0.9	1.14	0.92, 1.44	0.2

*CI* confidence interval, *CPP* cerebral perfusion pressure, *GCS* Glasgow Coma Score, *GOS-6* Glasgow Outcome Score at 6 months

## Discussion

In this multicenter, multinational study of childhood severe TBI requiring PICU care, we were the first to successfully produce the pediatric CPP dose–response plots visualizing a well demarcated transition curve separating the positive and negative associations between the occurrence of CPP insults and global outcome. This pediatric CPP dose–response plot was the first to demonstrate an exponential course similar to that seen in the adult TBI CPP dose–response study [22].

The adult CPP dose–response plot, using minute-by-minute “clinical grade” physiology data, clearly illustrated the intuitive concept that the lower the CPP, the shorter the time this could be tolerated by adult patients with TBI [22]. Although the ICP dose–response methodology was validated in pediatric TBI [19, 21], this current study is the first to successfully produce CPP dose–response plots in childhood brain trauma. Swedish colleagues attempted in 2023 to produce a pediatric CPP dose–response plot but were unsuccessful due to too few patients having GOS < 4 (14.75%), with CPP events excluded if they occurred in less than 20 patients (32.79%), making it near impossible determine CPP dose–response [32]. In contrast, 39 patients (37.5%) in the present study had GOS < 4, allowing us to produce the first pediatric CPP dose–response plot with red zones highlighting those CPP events associated with poor outcome, akin to the CPP dose–response concept previously described in adults [22].

Producing this first pediatric CPP dose–response visualization plot is important because it suggests that having a CPP below 56 mm Hg of any duration is associated with poor outcome in our patients. In contrast, the latest international pediatric TBI ICU treatment guidelines recommend targeting CPP between 40 and 50 mm Hg [17]. Efforts to optimize outcomes may require that CPP targets be higher than the insult thresholds identified in this study. The “critical” CPP insult threshold of 56 mm Hg observed in this current study is similar to that in previous childhood brain trauma studies [12, 36]. There is an urgent need to validate our pediatric CPP dose–response plot methodology to ascertain the lowest safe CPP target level for childhood TBI in a larger independent pediatric cohort. This will ensure stronger evidence-based recommendations for pediatric CPP treatment thresholds in the future and thus alleviate the existing uncertainty in the literature on what the minimum CPP target thresholds in children should be [7, 12, 36, 37].

The relationship between high CPP and outcome in pediatric TBI is poorly described in the literature, as previous studies have focused on the lowest CPP insult thresholds [12, 36, 37]. This current study demonstrated for the first time that high CPP was not completely harmless following pediatric TBI, that is, higher CPP was tolerated for a shorter duration [22]. This means both upper and lower transition curves for CPP dose–responses need to be considered together to produce the safe zone of CPP treatment targets in childhood TBI. This CPP safe zone concept concurs with a previous adult TBI CPP dose–response report [22]. Furthermore, cerebrovascular reactivity patterns affect the size of the safe zone for CPP in both adult [22] and pediatric TBI, as observed here. A recent article attempted to visualize high-frequency “research-grade” CPP, or ΔCPP_opt_ (actual CPP − calculated CPP_optimal_), data and outcome following adult TBI [38]. Despite including more than 500 adult patients’ data, they did not have sufficient data to produce the black transition curves separating good and poor outcome or identify the safe zone of CPP [38]. They did confirm low CPP was associated with worse outcome when cerebral autoregulation as measured by the pressure-reactivity index (PRx) was concurrently impaired [38].

We also demonstrated for the first time that ICP above 20 mm Hg was associated with poor outcome in pediatric patients with TBI regardless of CPP levels. This concurs with similar observations in the adult CPP dose–response study [22]. One explanation is that the high ICP reflects the seriousness of the patient’s whole clinical condition rather than the exact pressure level itself and therefore is reflected in the profound relationship with outcome. Although high ICP impacts post-TBI outcome, overruling the effects of CPP, this should not be interpreted as CPP management being unimportant during episodes of high ICP. Previous ICP dose–response studies in both adult and pediatric TBI highlighted ICP insults were not well tolerated if CPP was less than 50 mm Hg [19, 21].

Our current study has several limitations. The number of patients in this study is relatively small despite being a multinational, multicenter study involving 16 PICUs in seven countries. This confirms the reported decreased incidence of pediatric brain trauma in higher-income countries due to successful implementation of injury prevention initiatives [39, 40]. Due to the small sample size, especially those with poor outcome (GOS < 4), subgroup analysis to determine age effects on CPP dose and outcome in childhood TBI was not possible (Supplementary Fig. 5). Using global outcome as measured by GOS as our primary outcome is another important limitation of our study. This limits our ability to understand the impact of CPP dose–response on detailed functional outcome following childhood brain trauma. This is, however, a common weakness for multinational, multicenter studies due to the expensive detailed face-to-face neurodevelopmental assessments required for functional outcome assessments, which cannot be deployed at scale for multicenter studies.

Our analysis was conducted on observational study data obtained from a cohort of patients who received ICP- and CPP-directed treatments. This limits our ability to assess the individual contributions to clinical outcome of the secondary CPP and ICP insult intensity compared with the possible benefit or harm of medical interventions delivered to manage them.

All contributing centers for this study zeroed arterial blood pressure at the right atrial level, which is a routine clinical practice similar to other PICUs in the UK, Europe, and Australia. This methodological issue, arising from an observational clinical study, may introduce a potential source of error with the calculation of CPP due to varying hydrostatic pressure gradients. We attempted to quantify this issue by using our contributing centers’ data collected on patients’ trunk sizes and age, and all indicated that the error would tend more towards the lower end of the scale rather than the worst-case scenario of an adult-sized trunk measurement with 30 degrees head-up tilt. This is because the most common degree of head-up tilt employed in our cohort was 30 degrees in the majority (> 93%) of our patients, with the mean and median age in the region of 7 years old, and less than half of our patients were aged 12 years or older.

A trained researcher with prior PICU clinical experience and a custom-built automated artifact removal algorithm jointly completed the task of detecting and removing the obvious artifacts in the raw physiological data in this study. Using this joint approach to detect artifacts might potentially introduce bias, but we found that both the trained researcher and our custom-built automated algorithm successfully identified the artifacts as well as the independent experts in our previous studies [11, 12, 19, 21]. The trained researcher or the automated algorithm only removed the obvious artifacts, like our previous ICP dose–response study [21], but they did not detect less obvious artifacts because we wanted to retain as much of the granularity of the minute-by-minute data as possible.

## Conclusions

This first pediatric CPP dose–response plot produced a better insight into the impact of low and high CPP insults on outcome following childhood brain trauma, but our cohort size was insufficient for age-related subgroup analysis. CPP below 56 mm Hg was not tolerated in all pediatric patients with TBI in this study regardless of their vasoreactivity patterns. A safe zone of CPP was identified only in the cohort with active vasoreactivity pattern. Larger studies are required to validate CPP dose–response in pediatric TBI and to unravel age-specific CPP target thresholds.

## Supplementary Information

Below is the link to the electronic supplementary material.

Supplementary file1 (ZIP 7570 KB)

Supplementary file2 (DOCX 13 KB)

Supplementary file3 (DOCX 17 KB)

Supplementary file4 (DOCX 16 KB)
