# Supplementary figures and images for: KidsBrainIT: Visualization of the Impact of Cerebral Perfusion Pressure Insult Intensity and Duration on Childhood Brain Trauma Outcome
Source: Neurocrit Care. 2025 Jun 3;44(1):85–94. doi: 10.1007/s12028-025-02296-z (PMC12819434; doi:10.1007/s12028-025-02296-z)

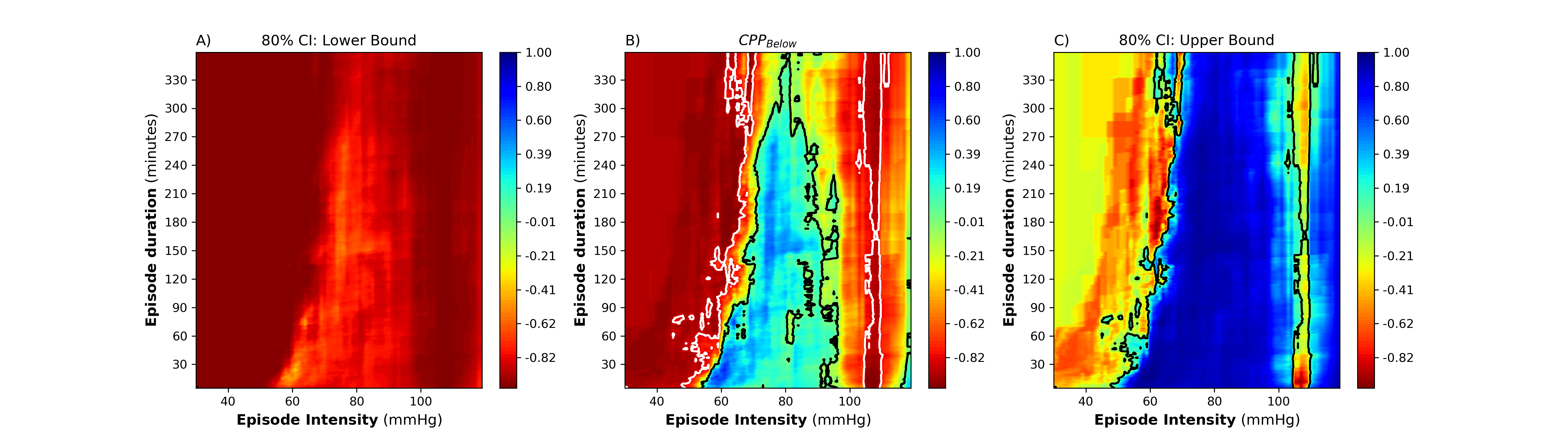

Supplement: Supplementary file 1 — Supplementary file1 (ZIP 7570 KB) [file 12028_2025_2296_MOESM1_ESM.zip › New folder/sFig1.tiff]

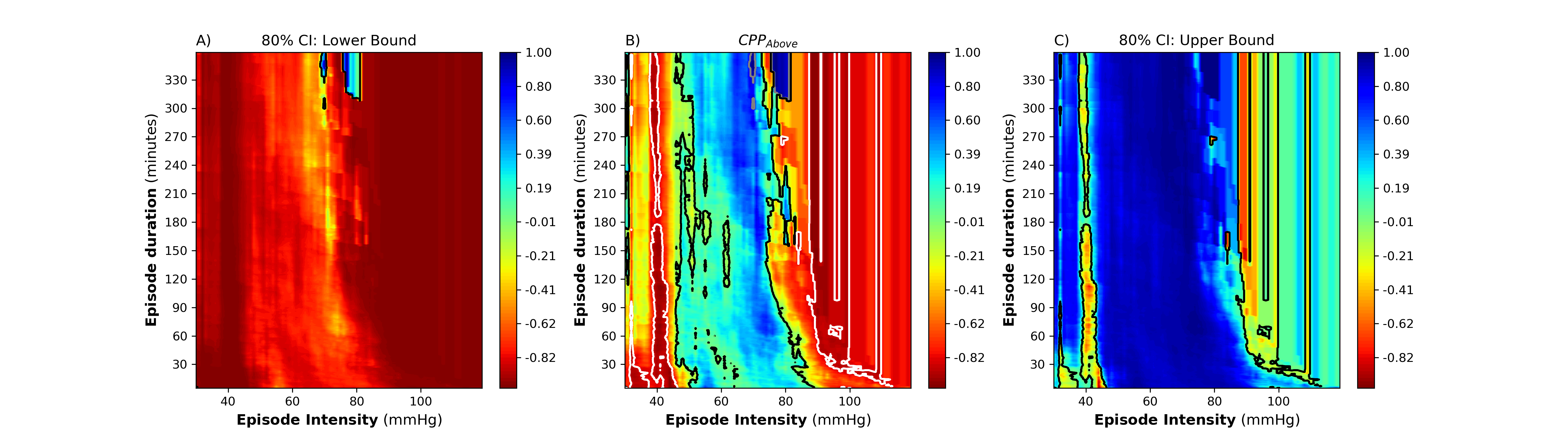

Supplement: Supplementary file 1 — Supplementary file1 (ZIP 7570 KB) [file 12028_2025_2296_MOESM1_ESM.zip › New folder/sFig2.tiff]

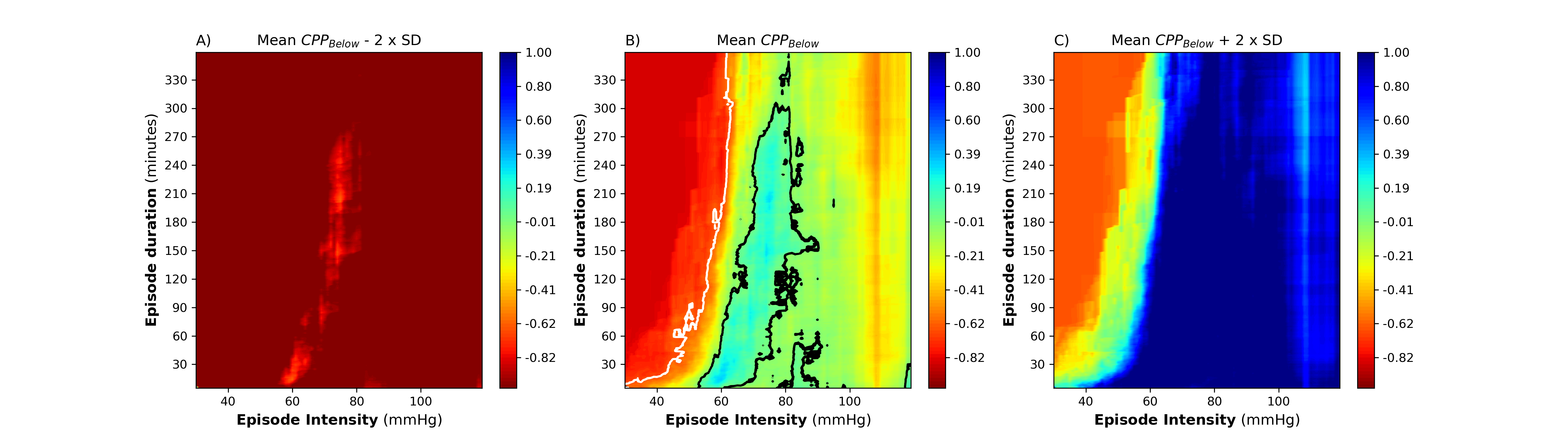

Supplement: Supplementary file 1 — Supplementary file1 (ZIP 7570 KB) [file 12028_2025_2296_MOESM1_ESM.zip › New folder/sFig3.tiff]

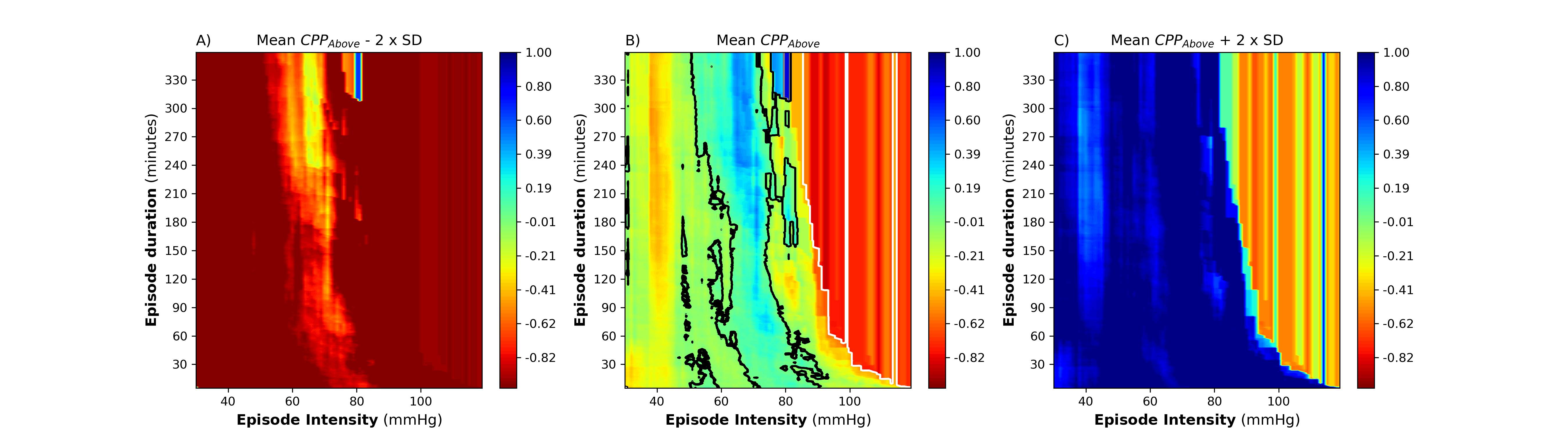

Supplement: Supplementary file 1 — Supplementary file1 (ZIP 7570 KB) [file 12028_2025_2296_MOESM1_ESM.zip › New folder/sFig4.tiff]

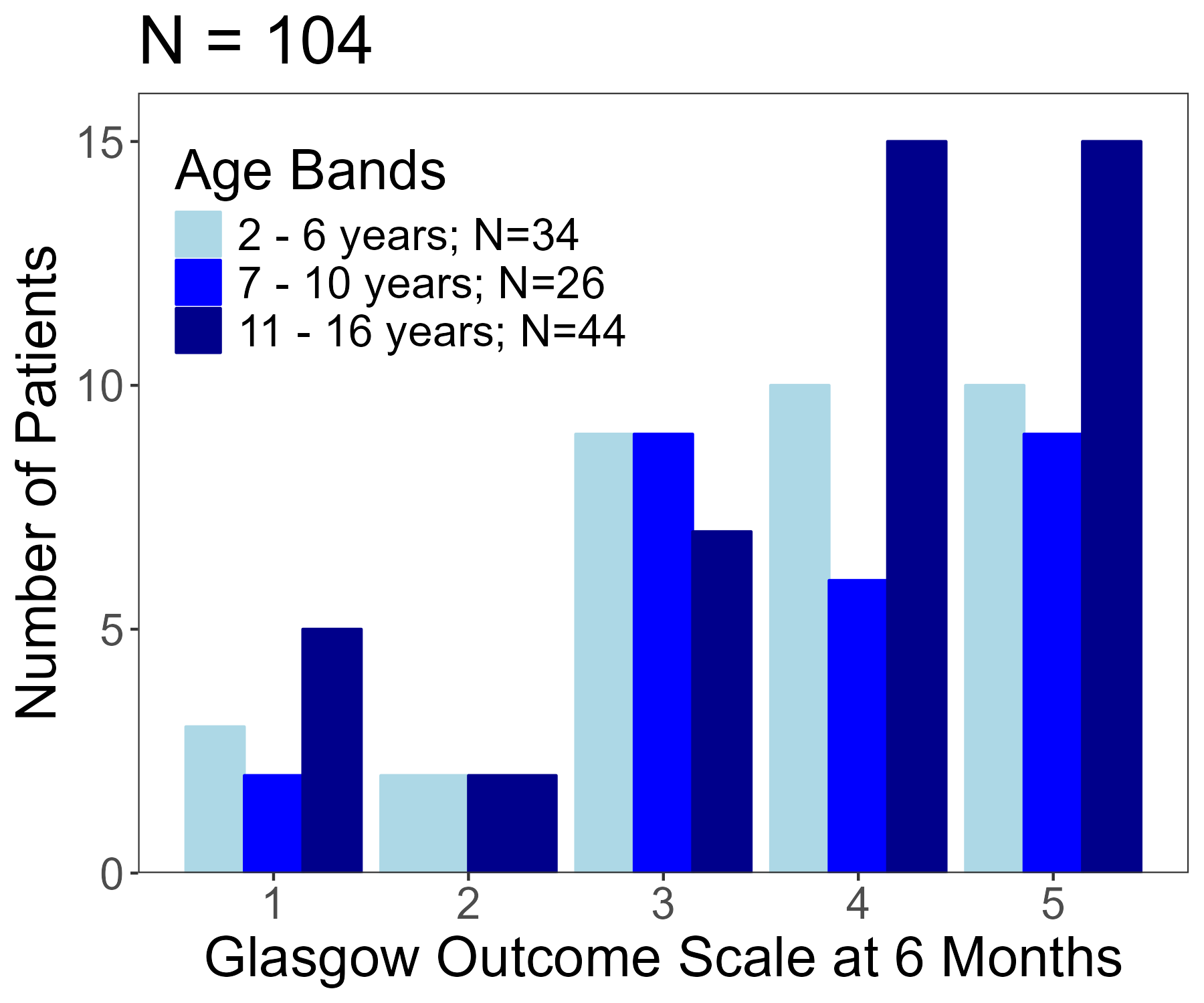

Supplement: Supplementary file 1 — Supplementary file1 (ZIP 7570 KB) [file 12028_2025_2296_MOESM1_ESM.zip › New folder/sFig5.tiff]
