# Supplementary material for: KidsBrainIT: Visualization of the Impact of Cerebral Perfusion Pressure Insult Intensity and Duration on Childhood Brain Trauma Outcome
Source: Neurocrit Care. 2025 Jun 3;44(1):85–94. doi: 10.1007/s12028-025-02296-z (PMC12819434; doi:10.1007/s12028-025-02296-z)
Supplement: Supplementary file 2 — Supplementary file2 (DOCX 13 KB) [file 12028_2025_2296_MOESM2_ESM.docx]

**Supplementary Figure Legends:**

**sFig. 1.** Original KidsBrainIT cerebral perfusion pressure (CPP) insult intensity - duration graph, $CPP_{Below}$, with an 80% confidence interval (CI) computed utilizing the Fisher transformation per calculated Pearson correlation. A) The contour plot of the 80% CI lower bound of all Pearson correlations present. C) the contour plot of the 80% CI upper bound of all Pearson correlations present. B) the original contour plot with the lower (grey) -and upper bound (white) transition lines, if present, added derived from the 80% CI.

**sFig. 2.** Original KidsBrainIT cerebral perfusion pressure (CPP) insult intensity - duration graph, $CPP_{Above}$, with an 80% confidence interval (CI) computed utilizing the Fisher transformation per calculated Pearson correlation. A) The contour plot of the 80% CI lower bound of all Pearson correlations present. C) the contour plot of the 80% CI upper bound of all Pearson correlations present. B) the original countour plot with the lower (grey) -and upper bound (white) transition lines, if present, added derived from the 80% CI.

**sFig. 3.** Cerebral perfusion pressure (CPP) insult intensity – duration graph, $CPP_{Below}$, after uniform population based bootstrapping (n = 1000) with replacement. A) Mean correlation matrix minus two times the standard deviation of the 1000 bootstrapped populations. C) Mean correlation matrix plus two times the standard deviation of the 1000 bootstrapped populations. B) The mean ICP insult intensity - duration graph with the lower (grey) -and upper (white) bound transition lines added, approximating a 95% confidence interval. Zones introduced in A) and C) where $\rho<-1$ or $\rho>1$ were set to -1 and 1, respectively.

**sFig. 4.** Cerebral perfusion pressure (CPP) insult intensity – duration graph, $CPP_{Above}$, after uniform population based bootstrapping (n = 1000) with replacement. A) Mean correlation matrix minus two times the standard deviation of the 1000 bootstrapped populations. C) Mean correlation matrix plus two times the standard deviation of the 1000 bootstrapped populations. B) The mean ICP insult intensity - duration graph with the lower (grey) -and upper (white) bound transition lines added, approximating a 95% confidence interval. Zones introduced in A) and C) where $\rho<-1$ or $\rho>1$ were set to -1 and 1, respectively.

**sFig. 5.** Histogram of age bands along Glasgow outcome score at 6 months of the KidsBrainIT population.
