# Supplementary material for: KidsBrainIT: Visualization of the Impact of Cerebral Perfusion Pressure Insult Intensity and Duration on Childhood Brain Trauma Outcome
Source: Neurocrit Care. 2025 Jun 3;44(1):85–94. doi: 10.1007/s12028-025-02296-z (PMC12819434; doi:10.1007/s12028-025-02296-z)
Supplement: Supplementary file 3 — Supplementary file3 (DOCX 17 KB) [file 12028_2025_2296_MOESM3_ESM.docx]

Suppl. Table 1: Time in Red Zone of CPPabove

|  | GOS Unfavourable | | | GOS Mortality | | |
| --- | --- | --- | --- | --- | --- | --- |
| **Characteristic** | **OR** | **95% CI** | **p-value** | **OR** | **95% CI** | **p-value** |
| Proportion Monitoring Time in Red Zone | 1.02 | 1.00, 1.05 | 0.069 | 1.04 | 1.00, 1.09 | 0.067 |
| Pupil Reactivity | 1.29 | 0.33, 5.31 | 0.7 | 0.92 | 0.05, 5.03 | >0.9 |
| Motor GCS | 0.69 | 0.50, 0.93 | 0.018 | 0.63 | 0.29, 1.05 | 0.13 |
| Age (years) | 1.00 | 0.89, 1.14 | >0.9 | 1.14 | 0.93, 1.44 | 0.2 |

^CPP, Cerebral Perfusion Pressure; GOS-6, Glasgow Outcome Score at 6 months; GCS, Glasgow Coma Score; OR, Odds Ratio; CI, Confidence Interval^
