# Supplementary material for: KidsBrainIT: Visualization of the Impact of Cerebral Perfusion Pressure Insult Intensity and Duration on Childhood Brain Trauma Outcome
Source: Neurocrit Care. 2025 Jun 3;44(1):85–94. doi: 10.1007/s12028-025-02296-z (PMC12819434; doi:10.1007/s12028-025-02296-z)
Supplement: Supplementary file 4 — Supplementary file4 (DOCX 16 KB) [file 12028_2025_2296_MOESM4_ESM.docx]

Suppl. Table 2: Time in Red Zone of CPPbelow

|  | GOS Unfavourable | | | GOS Mortality | | |
| --- | --- | --- | --- | --- | --- | --- |
| **Characteristic** | **OR** | **95% CI** | **p-value** | **OR** | **95% CI** | **p-value** |
| Proportion Monitoring Time in Red Zone | 1.01 | 0.97, 1.06 | 0.6 | 1.11 | 1.02, 1.23 | 0.021 |
| Pupil Reactivity | 1.34 | 0.36, 5.57 | 0.7 | 0.59 | 0.03, 3.90 | 0.7 |
| Motor GCS | 0.72 | 0.52, 0.95 | 0.029 | 0.68 | 0.31, 1.14 | 0.2 |
| Age (years) | 0.98 | 0.87, 1.10 | 0.7 | 1.07 | 0.88, 1.33 | 0.5 |

^CPP, Cerebral Perfusion Pressure; GOS-6, Glasgow Outcome Score at 6 months; GCS, Glasgow Coma Score; OR, Odds Ratio; CI, Confidence Interval^
